# Missing data imputation techniques for wireless continuous vital signs monitoring

**DOI:** 10.1007/s10877-023-00975-w

**Published:** 2023-02-02

**Authors:** Mathilde C. van Rossum, Pedro M. Alves da Silva, Ying Wang, Ewout A. Kouwenhoven, Hermie J. Hermens

**Affiliations:** 1 Biomedical Signals and Systems, University of Twente, Enschede, The Netherlands; 2 Cardiovascular and Respiratory Physiology, University of Twente, Postbox 217, 7500 AE Enschede, The Netherlands; 3 Department of Surgery, Hospital Group Twente, Almelo, The Netherlands; 4 NOVA School of Science and Technology, NOVA University of Lisbon, Lisbon, Portugal; 5 ZGT Academy, Hospital group Twente, Almelo, The Netherlands

**Keywords:** Vital signs, Physiological monitoring, Imputation, Missing data, Telemonitoring

## Abstract

Wireless vital signs sensors are increasingly used for remote patient monitoring, but data analysis is often challenged by missing data periods. This study explored the performance of various imputation techniques for continuous vital signs measurements. Wireless vital signs measurements (heart rate, respiratory rate, blood oxygen saturation, axillary temperature) from surgical ward patients were used for repeated random simulation of missing data periods (gaps) of 5–60 min in two-hour windows. Gaps were imputed using linear interpolation, spline interpolation, last observation- and mean carried forwards technique, and cluster-based prognosis. Imputation performance was evaluated using the mean absolute error (MAE) between original and imputed gap samples. Besides, effects on signal features (window’s slope, mean) and early warning scores (EWS) were explored. Gaps were simulated in 1743 data windows, obtained from 52 patients. Although MAE ranges overlapped, median MAE was structurally lowest for linear interpolation (heart rate: 0.9–2.6 beats/min, respiratory rate: 0.8–1.8 breaths/min, temperature: 0.04–0.17 °C, oxygen saturation: 0.3–0.7% for 5–60 min gaps) but up to twice as high for other techniques. Three techniques resulted in larger ranges of signal feature bias compared to no imputation. Imputation led to EWS misclassification in 1–8% of all simulations. Imputation error ranges vary between imputation techniques and increase with gap length. Imputation may result in larger signal feature bias compared to performing no imputation, and can affect patient risk assessment as illustrated by the EWS. Accordingly, careful implementation and selection of imputation techniques is warranted.

**Supplementary Information:**

The online version contains supplementary material available at 10.1007/s10877-023-00975-w.

## Introduction

With the evolution of mobile health technology, the use of wireless sensors for remote vital signs monitoring is rapidly increasing. In a hospital ward setting, wireless monitoring provides the opportunity to measure vital signs continuously, which allows active notification of vital signs abnormalities and evaluation of trends [1, 2]. Accordingly, remote technologies have been deployed to assist early identification of patient deterioration in high-risk surgical or general ward patients [3, 4], and were proposed for monitoring of isolated patients during the COVID-19 pandemic [5]. Furthermore, the continuous data can be used for automated analysis and risk modelling, aiming to support patient monitoring and clinical decision-making. Although standards for the analysis of continuous data in ward patients have not been established as of yet, the sensor data can, for example, be used for the objectification of trends over time based on signal characteristics or for automated calculation of early warning scores (EWS) that are currently used as part of rapid response systems in ward patients [2, 6]. Likewise, the vital signs measurements or extracted signal characteristics can be used as features for advanced event detection algorithms and (machine learning-based) risk prediction models that are increasingly being developed [7].

Despite the potential clinical benefits of remote continuous monitoring and corresponding risk modelling, the processing and interpretation of the data is still a major challenge and hampered by missing and poor quality data [8, 9], resulting in data loss of up to 50% [10, 11]. Measurement disturbances or disruptions are often caused by motion artefacts, which occur frequently during continuous wireless measurements in mobilizing patients [12, 13]. In addition, sensor malfunction or displacement and wireless connection issues can lead to artefacts or data loss [8, 14]. In case the missing or erroneous data periods are not corrected adequately, these segments will hinder the evaluation of vital signs abnormalities and trends. Furthermore, missing data segments will hamper feature extraction and thereby reduce the performance of event detection algorithms, acuity scores, or risk prediction models that are used for clinical decision-making [7, 13–16].

In current practice, retrospective imputation is often applied to substitute periods of missing data or removed erroneous segments in physiological time series data for further analysis or risk modelling. Traditionally, imputation is performed using basic methods such as carry forward techniques or replacement by the patient mean [17, 18]. These basic methods are easy to interpret in clinical practice, and therefore widely used. Yet, various alternative imputation methods that model the dynamic or personal characteristics of the data have been described more recently, which may be better suited for the evaluation of patterns or for personalized prediction models [16–19]. Although each imputation method has advantages and limitations, it is yet unclear how different imputation techniques perform when used for continuous vital signs monitoring in ward patients, and to what extent imputation could influence further analysis and clinical decision-making. Therefore, the current study aimed to evaluate and compare the performance of various techniques for retrospective imputation of missing data periods, and to explore the impact of imputation on patient monitoring by illustrating the effects on the extraction of basic signal features and calculation of early warning scores.

## Methods

### Data collection

The current study has a retrospective observational study design. Continuous vital signs recordings were obtained from an existing study database, including data from 60 adult patients that were admitted to the hospital ward for postoperative care after elective oesophageal or gastric surgery or hip fracture surgery in the Hospital Group Twente (ZGT, Almelo, the Netherlands) between 2018 and 2019. Vital signs were obtained every minute using wireless sensors connected to the Patient Status Engine (Isansys Lifecare Ltd., Oxfordshire, UK). The chest-worn LifeTouch sensor was used for measurements of heart rate (HR) and respiratory rate (RR), and the LifeTemp (Isansys Lifecare Ltd., Oxfordshire, UK) sensor was placed under the armpit to record axillary temperature (Temp). Blood oxygen saturation (SpO2) was measured with a finger probe attached to the wrist-worn Nonin WristOx2 3150 (Nonin Medical Inc., Plymouth, MN, USA). Measurements were performed in parallel to standard care. Both caregivers and patients were blinded for the continuously measured vital signs data. Correct functioning of the sensors was checked regularly during office hours, and measurements were re-established after sensor repositioning, if needed. All data was uploaded to MATLAB (MathWorks, Inc.) for further analysis and simulation. Vital signs recordings were preprocessed by removing values that exceeded the expected physiological range [20] (HR > 200 or < 30 bpm, RR > 50 or < 5 brpm, SpO2 < 70%, Temp > 50 or < 30 °C). Likewise, samples reporting error codes provided by the system in case of measurement interruptions caused by sensor displacement or disconnection were removed. Furthermore, a 4 min window-based median filter was applied [21].

### Data loss evaluation

To explore the degree of missing data in the current database and thereby evaluate the clinical relevance of data imputation, the percentage of the total recording time where one-minute vital signs samples were missing before and after preprocessing was calculated. In addition, the amount and duration of missing data periods were assessed for each vital parameter. Interruptions longer than 4 h were not included in this count, as these comprise a major part of an eight-hour nurse shift and were therefore not regarded as part of continuous measurements.

### Missing data simulation

**Fig. 1:**
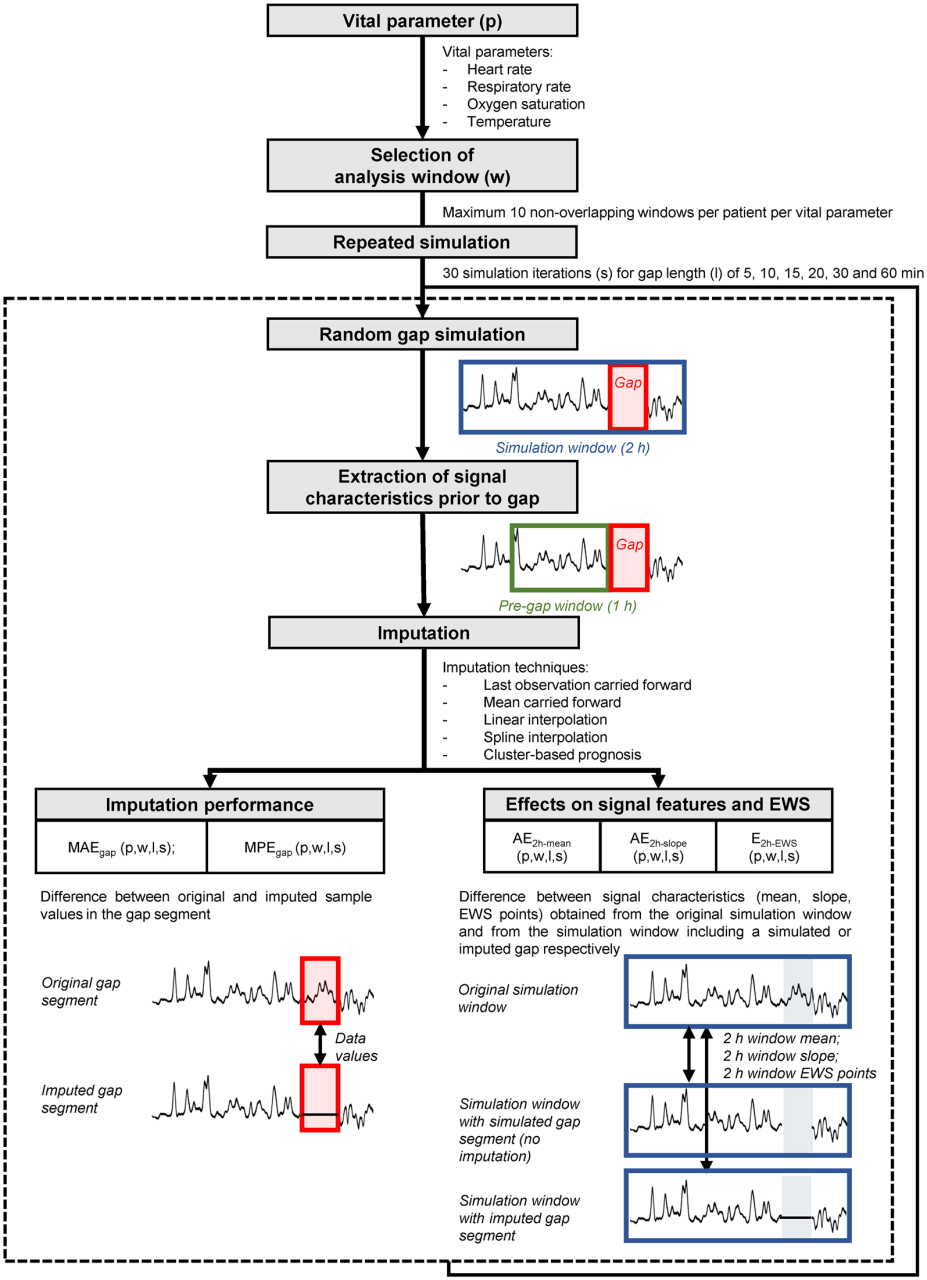
Overview of the missing data simulation process and evaluation of imputation techniques. *MAE*_*gap*_ mean absolute error of the imputed gap segment, *MPE*_*gap*_ mean percentage error of the imputed data gap, *AE*_*2h-mean*_ absolute error of the mean value of the two-hour simulation window, *AE*_*2h-slope*_ absolute error of the slope of the two-hour simulation window, *E*_*2h-EWS*_ error of the EWS points assigned to the two-hour simulation window, *EWS*: early warning score, *p* parameter, *w* window, *s* simulation iteration, *l* gap length

**Fig. 2:**
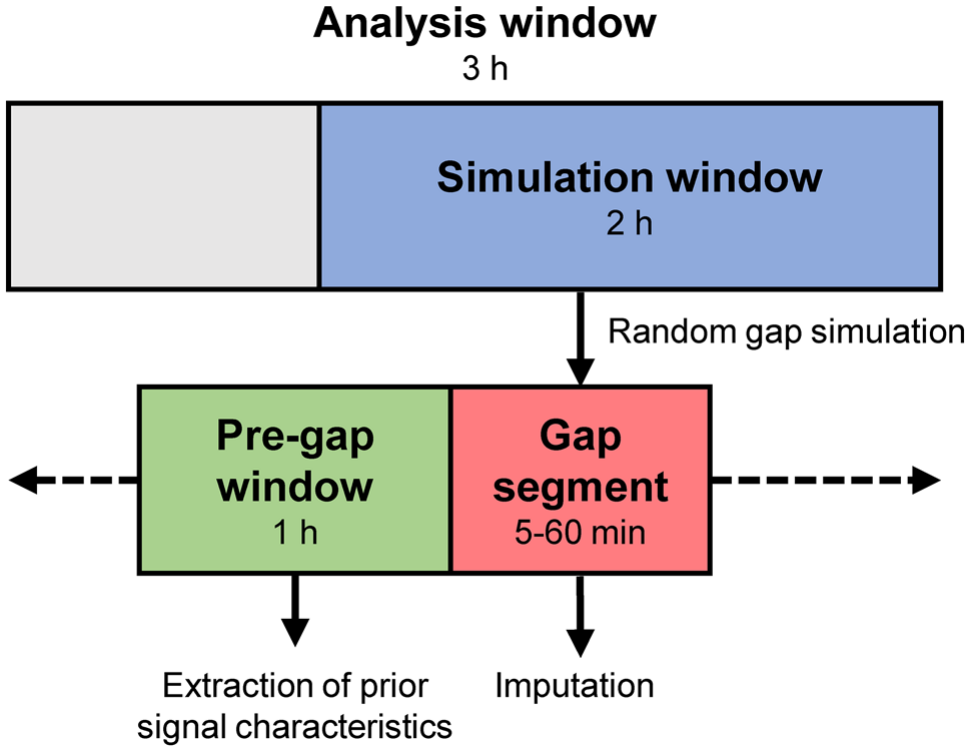
Illustration of the windows used in the gap simulation process. In each iteration of the simulation process, a missing data period (gap segment) of a predefined length (5, 10, 15, 20, 30, or 60 min) is generated at random within the simulation window and used to test imputation techniques. The pre-gap window is used to extract signal characteristics prior to the gap segment

Missing data periods (‘gaps’) were simulated in real uninterrupted continuous vital signs recordings to evaluate the performance of different imputation methods. Figure 1 provides an overview of the main steps of the simulation and evaluation process. In each patient, a maximum of ten windows of three hours each was selected for analysis for each of the vital signs (‘analysis window’). Analysis windows were selected subsequently using a sliding window approach, allowing no overlap. Furthermore, windows were only selected in case the concerning vital sign measurement did not contain any missing values. The last two hours of each window was allocated as ‘simulation window’ and used for simulation of gap segments (Fig. 2). This simulation window size was selected based on the assumption that—although there is no consensus regarding the optimal monitoring frequency [22]—the (average) vital signs values would ideally be updated at least every two hours to enable evaluation of the risk level of ward patients which typically deteriorate in a period of hours [23]. Gap segment simulation was performed by randomly generating one artificial period of missing data within the simulation window. Simulation was repeated 30 times per simulation window, and for gap segment lengths of 5, 10, 15, 20, 30, and 60 min, respectively. For each simulated gap segment, the one-hour window preceding the gap was assigned as the ‘pre-gap window’, which was used for extraction of prior data characteristics by some of the imputation techniques.

### Imputation techniques

**Fig. 3:**
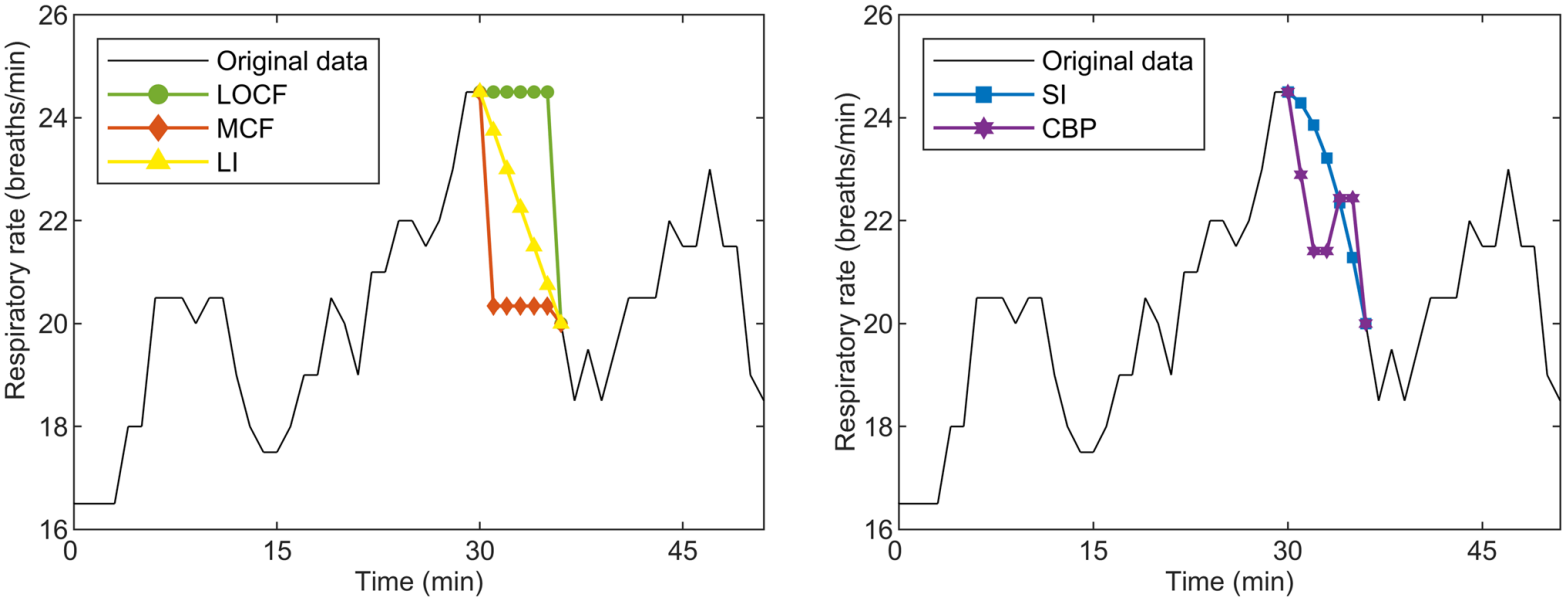
Illustration of the imputation of missing data by different imputation techniques. *LOCF* last observation carried forward, *MCF* mean carried forward, *LI* linear interpolation, *SI* spline interpolation, *CBP* cluster-based prognosis

Five different imputation techniques were tested, including the last observation carried forward (LOCF), mean carried forward (MCF), linear interpolation (LI), and spline interpolation (SI) techniques [24], and a cluster-based prognosis technique (CBP). The first four methods were selected because these represent traditional and basic imputation methods that are widely used for physiological signal processing and imputation of vital signs [17, 18, 25–30], whereas the last method was selected to explore a more advanced technique performing personalized estimation of vital sign patterns [31]. The differences in imputation techniques are illustrated in Fig. 3.

The LOCF technique substitutes all samples in the gap segment by the last sample value prior to the data gap. The MCF technique is a variant of the LOCF method, aiming to estimate the missing data based on a longer measurement period. Accordingly, the MCF technique uses the mean value of the one-hour pre-gap window to fill the gap segment. In the LI technique, the gap segment is substituted by a linear function, which is estimated using the latest sample value prior to the data gap and the first sample value after the gap. Similarly, the SI technique imputes the gap segment with a cubic spline function. The CBP technique is adapted from imputation methods described by Sun et al. [31], where a regression model is used to impute missing data using similar data segments obtained in similar patients. Details of the CBP technique and modifications that were made as compared to Sun’s method are described in Supplementary file 1.

### Performance evaluation

The performance of each imputation technique was assessed using the mean absolute error (MAE) and mean percentage error (MPE). The MAE and MPE were calculated for each simulated gap by respectively averaging the absolute or relative difference between the imputed data value (${\widehat{x}}_{i}$) and corresponding original data value (${x}_{i}$) for all data samples ($i$) in the gap segment with length $l$, following Eqs. 1 and 2:

1 $${MAE}_{gap}=\frac{\sum _{i=1}^{l}\left|{x}_{i}-{\widehat{x}}_{i}\right|}{l}$$

2 $${MPE}_{gap}=\frac{\sum _{i=1}^{l}\left|\frac{{x}_{i}-{\widehat{x}}_{i}}{{x}_{i}}\right|}{l}*100\%$$

As simulation was performed 30 times per analysis window for all combinations of simulated gap length and vital parameters, the MAE_gap_ and MPE_gap_ were averaged across these iterations to obtain the results per analysis window for each of these combinations. The MAE_gap_ values of all analysis windows were evaluated separately for the different gap segment lengths and different vital parameters, to evaluate the range of performance for each imputation technique. The MPE_gap_ was used to explore differences in overall performance between imputation techniques and between vital parameters.

Last, for each vital parameter, the median MAE_gap_ of all simulations performed in assessment windows with 10% lowest and 10% highest original mean value were compared with the median MAE_gap_ of the remaining windows, aiming to explore the influence of vital sign levels on imputation performance. Likewise, the MAE_gap_ was compared for assessment windows with highest and lowest standard deviation to investigate the effect of data variability.

### Clinical impact exploration

#### Effects on signal features

In clinical practice, the evaluation of vital signs measurements by caregivers does not only rely on individual vital signs values but also involves evaluation of vital signs trends, i.e., whether vital signs are stable or increase or decrease over time [32]. Although there is still little evidence regarding the clinical value of automated trend assessment methods for vital signs monitoring, studies have indicated that basic trend metrics such as the average value or slope can contribute to clinical risk prediction models [33, 34]. To explore to which extent imputation may influence the extraction of signal features that could be relevant for trend identification or risk modelling, we compared the mean value and linear slope of the two-hour simulation window before and after imputation. Accordingly, the absolute error (AE) between the mean value of the original two-hour simulation window and the mean value of the simulation window with an imputed gap segment was calculated. resulting in the AE_2h − mean_. In addition, the AE_2h − mean_ was also calculated for the simulation window after deletion of the gap samples, i.e., following an available-case analysis approach, which served as a reference for trend estimation without imputation. Like the AE_2h − mean_, the absolute error was also computed for the slope (AE_2h − slope_), for all imputation techniques, and for the situation without imputation. For the AE_2h − slope_, windows with an original absolute slope value < 0.0025 per hour were excluded as the slope feature was considered clinically irrelevant for stable measurements.

#### Effects on early warning scores

Early warning scores (EWS) are used widely in clinical wards to assess the risk of patient deterioration. Although many variants exist, the EWS is obtained by assigning points for every vital sign, where the number of points increases for larger deviations from their normal range. The EWS is calculated as the sum of all assigned points and used to trigger further patient assessment or care escalation in case the total EWS exceeds a pre-set threshold [6]. Although vital sign measurements currently rely on nurse observations, there is growing interest to use sensor technologies for (partial) automation of EWS measurements [2]. To investigate the possible consequences of imputation on the EWS, we investigated for each vital parameter to what extent the points assigned to the vital parameters obtained from the sensor recordings were affected by imputation. Accordingly, for each simulation, the mean value of the two-hour assessment window was categorized according to the criteria described in Table 1 before and after imputation. The criteria of HR, RR, and Temp were based on the Modified Early Warning Score (MEWS), which is widely used [13]. As SpO2 is not included in the MEWS, the SpO2 criteria were obtained from the National Early Warning Score (NEWS) criteria [18]. For each parameter, the error (E_2h − gap_) between the points assigned to the original window and the window after gap simulation or imputation was assessed. Correspondingly, the number of simulations which resulted in misclassification of the EWS (i.e., E_2h − gap_ ≠ 0) was calculated.

**Table 1:** Criteria for early warning score (EWS)

Parameter	EWS points						
	3	2	1	0	1	2	3
Heart rate (bpm)		≤ 40	41–50	51–100	101–110	111–129	≥ 130
Respiratory rate (brpm)		≤ 8		9–14	15–20	21–29	≥ 30
Oxygen saturation (%)	≤ 91	92–93	94–95	≥ 96			
Temperature (°C)		≤ 34.9		35-38.4		≥ 38.5	

## Results

### Data collection

The database included vital signs recordings obtained from 60 hospitalized post-surgical patients, of which 8 patients were excluded due to incomplete demographical data. A total of 52 patients were included, of which 15 patients experienced one or more complications (Clavien Dindo Class I–III) during the monitoring period. The demographics of the included patients are reported in Table 3 (Supplementary file 2).

### Data loss

**Fig. 4:**
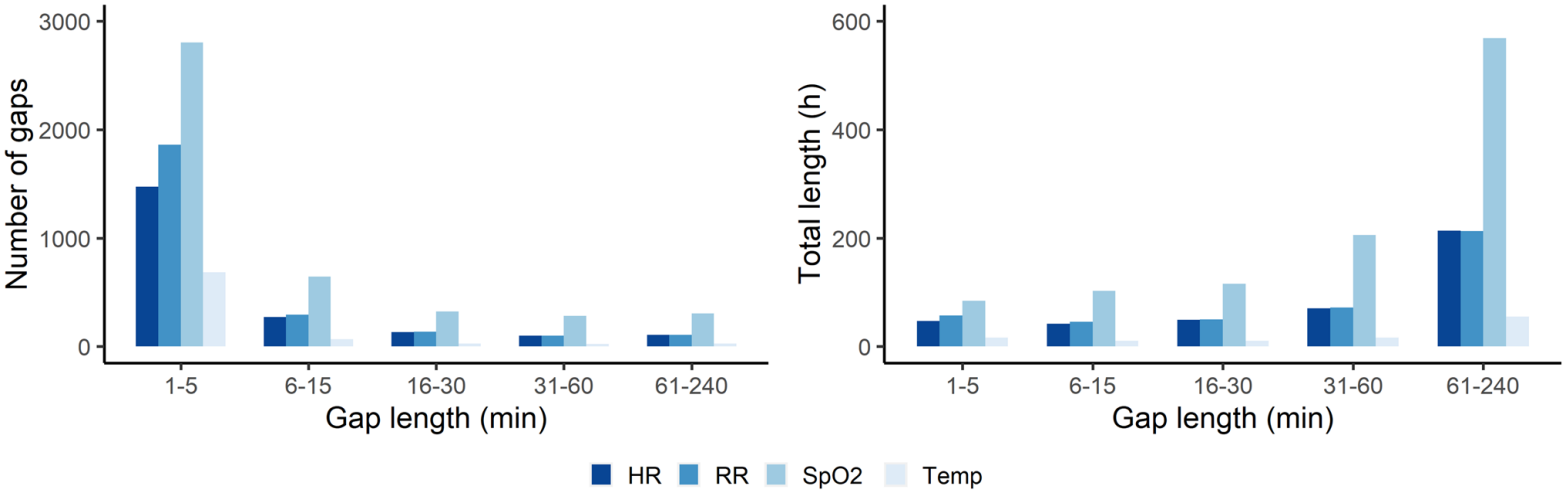
Number and total length of missing data periods (gaps) with a gap length of up to 4 h observed in the vital signs recordings of the included patient population. *HR* heart rate, *RR* respiratory rate, *SpO*2 blood oxygen saturation, *Temp* temperature

The original dataset of included patients contained vital signs recordings with a median duration of 119 h (IQR: 93–147) per vital sign, resulting in a total of 6792 h of monitoring data. The median data availability in these recordings was 86% (IQR: 72–94%) for HR, 86% (IQR: 72–94%) for RR, 46% (IQR: 38–61%) for SpO2, and 96% (IQR: 81–99%) for Temp. In total, 0.2% of the missing data was related to outlier removal whereas 60% was related to sensor displacement or disconnection as reported by the system. For the remaining missing samples, data was missing without further information. Figure 4 reports the number and total duration of missing data periods up to 4 h that was observed in the original dataset. Most of the gaps that were observed had a duration of 1–5 min, whereas larger gaps were observed less frequently. Nevertheless, the total duration of larger gaps was higher compared to short data gaps.

### Missing data simulation

From the original data recordings, a total of 1743 three-hour analysis windows (497 for HR, 492 for RR, 264 for SpO2, 490 for Temp) were eligible for simulation, with a median of 34 (IQR: 31–39) windows per patient. As gap simulation was repeated 30 times for each gap size in every analysis window, a total of 313,740 gaps were simulated.

### Performance evaluation

**Fig. 5:**
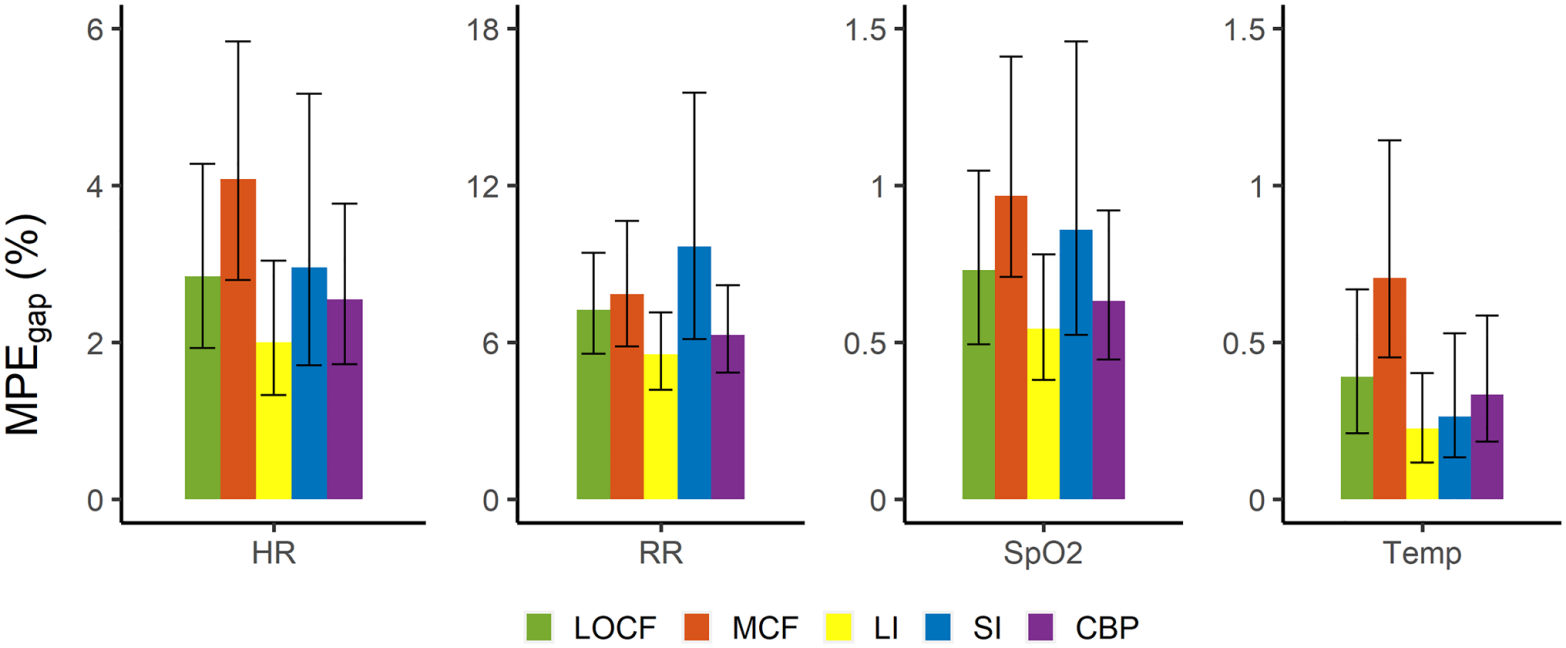
Mean Percentage Errors (MPE_gap_) observed for imputation of missing data periods simulated in individual vital signs. The MPE_gap_ is shown as median with interquartile range, and described results found for all simulated gap lengths. *LOCF* last observation carried forward, *MCF* mean carried forward, *LI* linear interpolation, *SI* spline interpolation, *CBP* cluster-based prognosis, *HR* heart rate, *RR* respiratory rate, *SpO*2 blood oxygen saturation, *Temp* temperature

Figure 5 reports the MPE_gap_ observed across all gap lengths for each parameter. For the HR, RR, and SpO2, the median MPE_gap_ and corresponding upper quartile ranges were lowest for the LI technique followed by the CBP and LOCF techniques, but interquartile ranges were relatively large and overlapping. The median and upper quartiles of the MPE_gap_ were highest for the MCF and SI methods. The same performance ranking was found for Temp, except for the fact that SI showed the second lowest median MPE_gap_. Comparing results between vital parameters, MPE_gap_ ranges were largest for the RR with median MPE_gap_ ranging between 5.5% for LI to 9.7% for SI, followed by the HR (2.0% for LI to 4.1% for MCF), SpO2 (0.5% for LI to 1.0% for MCF) and Temp respectively (0.2% for LI to 0.7% for MCF).

**Fig. 6:**
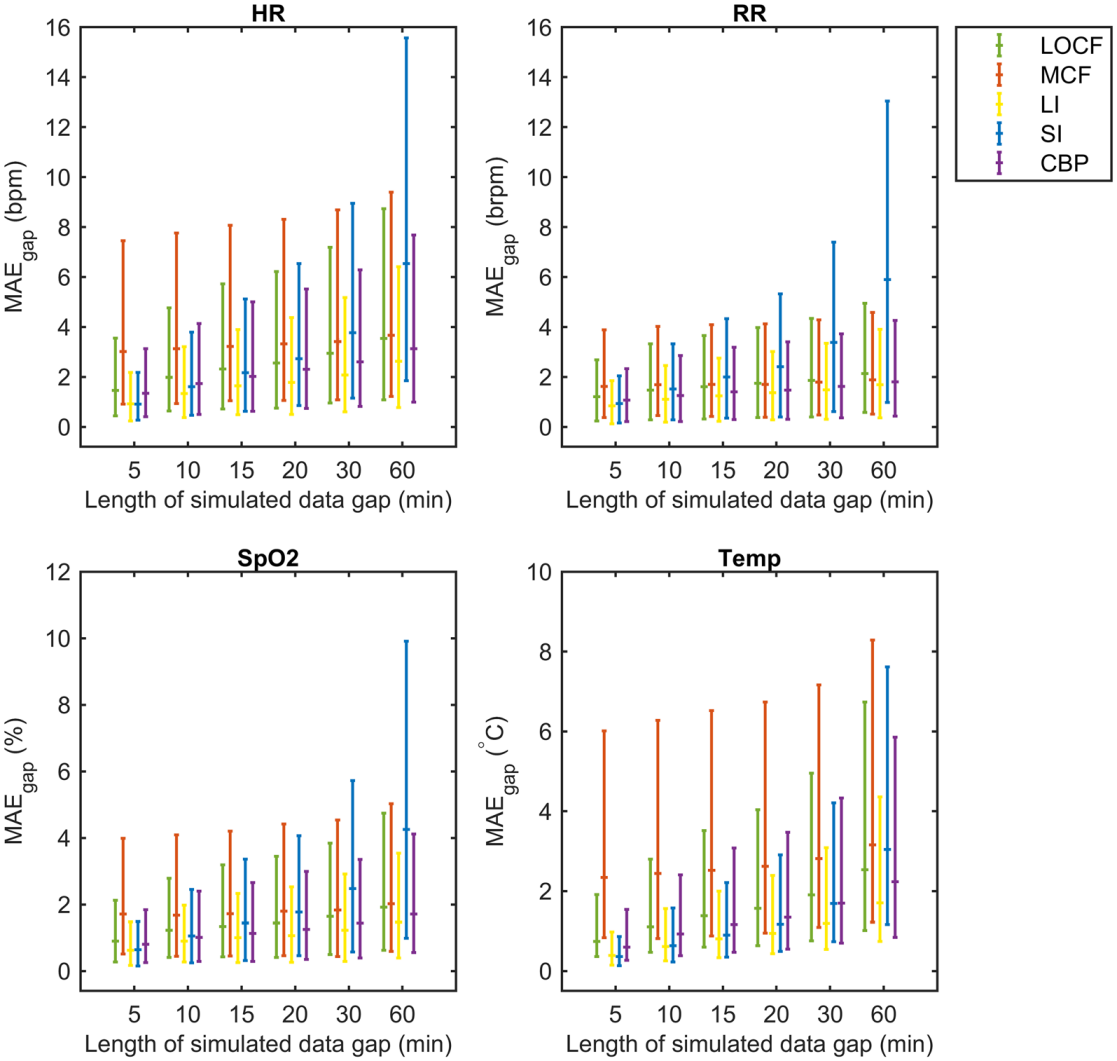
Mean Absolute Error (MAE_gap_) observed for imputation of simulated missing data periods (gaps) of 5–60 min length. The MAE_gap_ is shown as median with interquartile range. *LOCF* last observation carried forward, *MCF* mean carried forward, *LI* linear interpolation, *SI* spline interpolation, *CBP* cluster-based prognosis, *HR* heart rate, *RR* respiratory rate, *SpO*2 blood oxygen saturation, *Temp* temperature

Looking at the absolute errors across different gap sizes (Fig. 6), MAE_gap_ ranges increased with gap size for all vital parameters, in particular for the SI method. The order of performance was similar as found for the MPE_gap_ results, where LI showed the lowest median MAE_gap_. The MAE_gap_ of the LI technique for gaps of 5 to 60 min ranged from HR: 0.9–2.6 bpm, RR: 0.8–1.8 brpm, SpO2: 0.3–0.7%, and Temp: 0.04–0.17 °C. For small gap sizes, highest error rates were typically found for MCF whereas large gap sizes showed highest errors for SI. The median MAE_gap_ reached values up to 6.5 bpm (SI technique) for the HR, 5.9 brpm for RR (SI technique), 2.1% for SpO_2_ (SI technique), and 0.31 °C for Temp (MCF technique) for gaps of 60 min.

Supplementary file 3 reports the MAE_gap_ ranges for all simulations performed in the assessment windows with 10% lowest and 10% highest mean value or standard deviation, respectively. For the HR and RR, the median MAE_gap_ and interquartile ranges were largest for windows with the highest mean value, and lowest for windows with lowest mean, whereas the opposite effect was observed for the SpO2 and Temp. For all vital parameters, MAE_gap_ ranges were lowest for windows with the lowest standard deviation and highest for the windows with the highest standard deviation. MAE_gap_ varied most between assessment window clusters for the MCF method, followed by the LOCF method.

### Clinical impact exploration

#### Effects on signal features

**Fig. 7:**
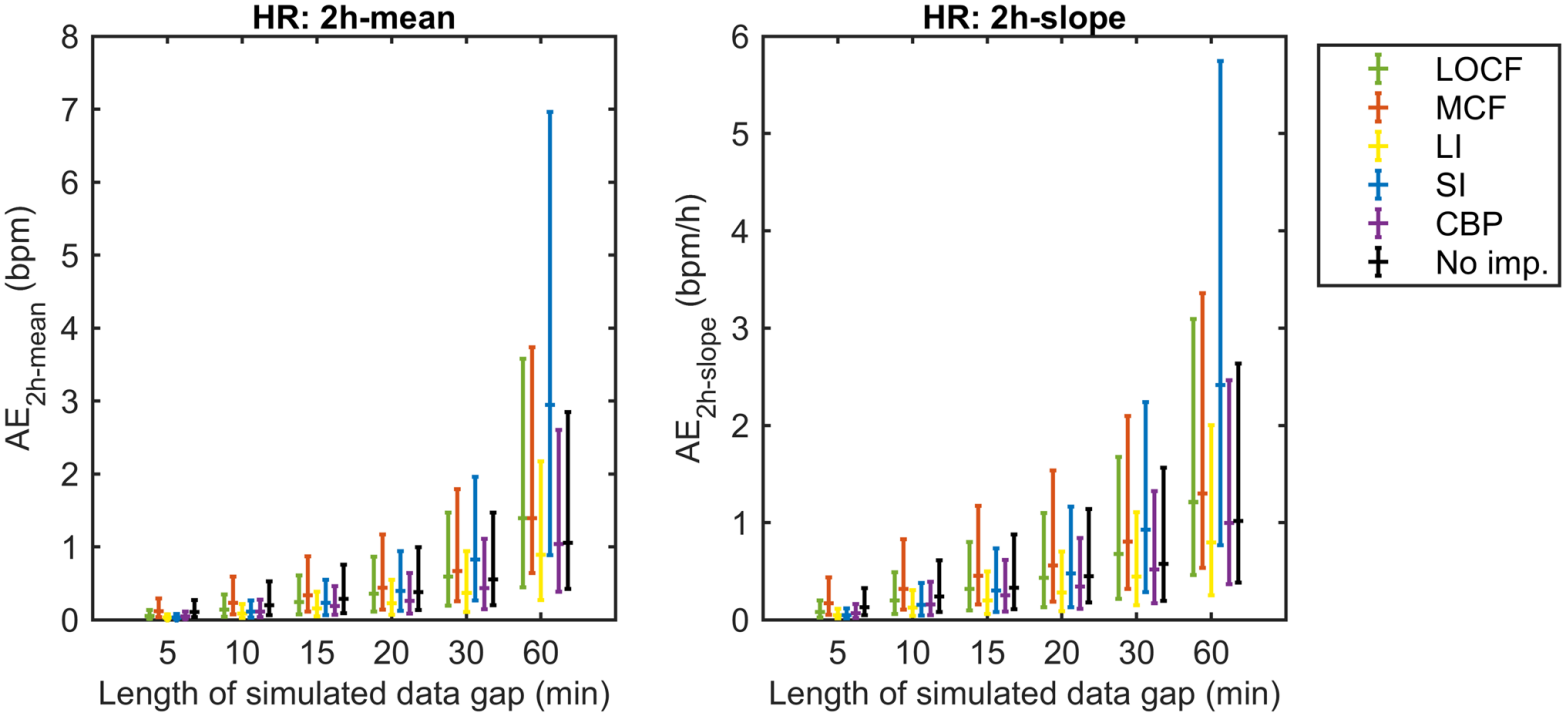
Absolute error of the mean value (AE_2h-mean_) and the slope (AE_2h-slope_) of the two-hour simulation window found for the heart rate (HR). The absolute error is shown as median with interquartile range for different imputation techniques and for the situation without imputation. *LOCF* last observation carried forward, *MCF* mean carried forward, *LI* linear interpolation, *SI* spline interpolation, *CBP* cluster-based prognosis, *No imp*. no imputation,

The AE_2h − mean_ and AE_2h − slope_ obtained by comparing the mean value and slope of the simulation window before and after simulation are shown in Fig. 7 for the HR, and in Supplementary file 4 for RR, SpO2 and Temp. As for the MAE_gap_, the AE_2h − mean_ and AE_2h − slope_ increased with gap segment length. Comparing estimations of the two-hour window mean, the median AE_2h − mean_ and upper quartiles were lowest for the LI or CBP techniques for all gap sizes, although interquartile ranges highly overlapped with other techniques. For the slope, the LI technique was associated with the lowest median AE_2h − slope_ for almost all gap sizes, ranging between 0.05 and 0.8 bpm/hour for HR, 0.04–0.5 brpm/hour for RR, 0.00–0.08%/hour for SpO2 and 0.02–0.23 °C/hour for Temp for gaps of 5–60 min. Comparing trend estimations after imputation to estimations based on non-imputed data, the median AE_2h − mean_ and AE_2h − slope_ of the LI and CBP method and corresponding upper quartiles were lower as compared to performing no imputation for almost all gap sizes in all vital parameters. In contrast, in comparison to no imputation, median AE_2h − mean_ and AE_2h − slope_ and upper quartiles were larger for the highest gap size(s) for the LOCF and SI, and for all gap sizes for the MCF technique.

#### Effects on early warning scores

**Fig. 8:**
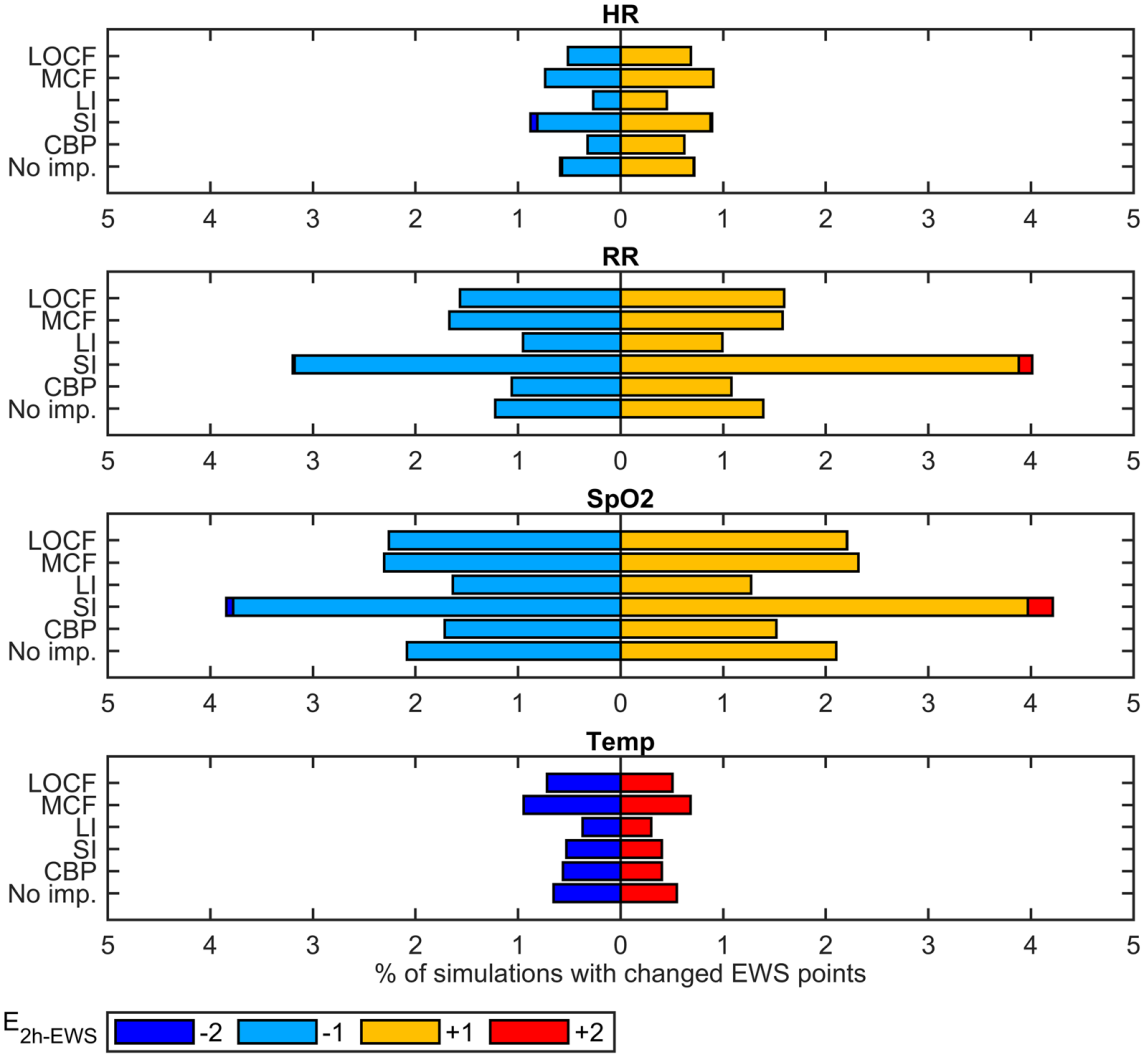
Percentage of simulations where (no) imputation led to a different number of EWS (early warning score) points assigned to individual vital parameters, compared to the original data. The colors present the error of the EWS points (E_2h-EWS_), indicating an increase (+ 1 or + 2 points) or decrease (−1 or −2 points) in EWS points. *LOCF* last observation carried forward, *MCF* mean carried forward, *LI* linear interpolation, *SI* spline interpolation, *CBP* cluster-based prognosis, *HR* heart rate, *RR* respiratory rate, S*pO*2 blood oxygen saturation, *Temp* temperature

Figure 8 presents the percentage of simulations performed in each parameter where the EWS was misclassified (i.e., E_2h − gap_ ≠ 0) after gap simulation and imputation respectively. Overall, imputation led to different EWS points in 1–2% of all simulations for HR and Temp, and between 2 and 7% for RR and 2–8% for SpO2. Changes were observed in both directions, where the number of simulations with increased points was comparable with the number of simulations with decreased points. In most cases, the EWS increased or decreased one level, resulting in E_2h − EWS_ of ± 1 points for HR, RR, and SpO2 and ± 2 points for Temp (see Table 1). Similar to the results presented for the extraction of signal features, imputation using the LI and CBP techniques had a lower impact on EWS calculation compared to performing no imputation, whereas the LOCF, MCF, and SI methods showed more or higher changes in EWS points for several parameters.

## Discussion

### Main findings

This study explored the performance and related clinical impact of various techniques for imputing missing data periods in continuous vital signs recordings obtained using wearable wireless sensors in postoperative surgical patients. The results indicated that the performance of imputation techniques varied largely between simulation windows, and that imputation errors strongly increased with gap segment length. Of all vital parameters, imputation had the most impact on respiratory rate measurements as suggested by the percentage error rates. Although the error ranges found for the different imputation techniques overlapped, we observed structural differences between the median errors and corresponding interquartile ranges. The LI technique resulted in the lowest median errors and smallest error ranges compared to the other imputation techniques. The largest median errors and error ranges were observed for the SI and MCF techniques. Similar results were found for the signal features extracted from the two-hour simulation window, where error ranges varied between and within vital parameters, techniques, and gap lengths. The LI and CBP techniques led to lower median bias and a smaller interquartile range of the windows’ slope and mean as compared to the deletion of missing data periods. In contrast, however, the MCF, SI, and LOCF techniques were associated with a larger (range of) bias compared to performing no imputation for most gap sizes. Therefore, these techniques can have adverse effects on the accuracy of signal features, and create most uncertainty in further analysis. Imputation led to an increase or decrease in the number of EWS points assigned to vital parameters in up to 8% of all simulations, which illustrates that imputation can affect clinical decision-making.

### Implications

Missing data is a relevant issue in remote vital signs monitoring in ward patients, as observed by the large missing data rates observed in the present study and other studies [10, 11]. Although most data gaps observed in the original recordings had a short duration, larger gaps contributed most to the total duration of missing data, which indicates that imputation is relevant for gaps of variable lengths. The current study highlights the importance of careful implementation and selection of imputation techniques, as error rates strongly varied between and within techniques, in particular for larger gap sizes.

Although the performance ranges of imputation techniques overlap, LI is suggested as the preferred method for retrospective imputation since this method showed the lowest median error rates and corresponding interquartile ranges and therefore brings the lowest risks of high error rates. Furthermore, this method is simple and therefore relatively easy to implement and intuitively understood by clinicians. This finding is in line with other studies reporting that linear interpolation generally provides higher imputation accuracy in vital signs data compared to other methods [18], and improves the performance of classification models based on physiological data [16]. The CBP technique showed the second-best performance for most parameters. As the CBP technique relies on model training, it can be expected that the performance of this technique will improve with further model optimization using larger datasets tailored to the population of interest. Since the CBP method estimates the dynamical characteristics of the missing data, this or similar personalized approaches may thereby be considered for intelligent models [15, 16].

In the investigation of the window slope and mean, we observed lower median errors and corresponding upper quartiles, compared to performing no imputation for the LI and CBP methods. Therefore, these techniques can improve the accuracy of signal feature extraction in measurements containing missing data periods and reduce the uncertainty in further data analysis. Conversely, we observed that the MCF, LOCF, and SI techniques were associated with larger error ranges as compared to performing no imputation for some or all gap lengths and resulted most often in EWS misclassification. A possible explanation for these observations is that these methods do not (adequately) estimate the variability of data estimations and are affected most by outliers prior to or after the data gap. Correspondingly, we observed that signal variability had the most influence on error rates in these methods. Therefore, we do not recommend using these techniques for retrospective imputation. These findings are of clinical relevance, as the LOCF and MCF or similar imputation methods are commonly applied for vital signs imputation in early warning scores or other risk prediction models [25–29].

Independent of the technique that is selected, one should be aware that imputation by definition results in data uncertainty, where the possible benefits—compared to performing no imputation at all—but also the risks for clinical decision-making will depend on the size and variability of errors. The median percentage of errors found across all simulations remained below 10% for each vital parameter, which indicates that the clinical risks of imputation are limited in most cases. Correspondingly, the risk that imputation affects the EWS points assigned to individual parameters was 1–8%, which could be reasonable in non-acute settings. On the other hand, the performance of the imputation techniques varied considerably between simulation windows, as reflected by the large interquartile ranges, creating uncertainty for further risk modelling. Besides, the relatively high upper quartiles indicate that there is a considerable risk of large imputation errors, in particular for larger gap sizes. Last, it is likely that missing data periods will be present simultaneously in multiple vital parameters, since measurements often rely on the same sensor or data connection. In this case, the uncertainty of risk models that rely on multiple parameters—such as the EWS—will increase even more. For some clinical applications, these (risks of) high errors are unacceptable, for example when it compromises safety by underestimating risk in unstable patients. As such, it is highly important to assess when the use of imputation is no longer justified.

In practice, the clinical team has to decide which level of uncertainty is acceptable for which patient, and for how long. Obviously, the clinical condition of the patient and corresponding suspicion for deterioration is paramount, as this defines the required level of monitoring. For example, for patients that have been stable for 2 days and are nearing hospital discharge, it will suffice if the care team evaluates general vital sign trends or the risks computed by computer models only once every nurse shift. In these patients, the imputation of gaps of up to one hour could be acceptable, as the overall risks for clinical decision-making and patient safety will be limited. However, patients that have just been discharged from the intensive care unit are often less stable and have a larger risk of serious deterioration. Accordingly, vital sign levels and patient risks need to be assessed more frequently and with higher accuracy levels, as small vital deviations could be critical. In these cases, it can be decided to allow imputation only for data containing short gaps to restrict the uncertainty of data and corresponding decisions, especially because imputation errors seem to be larger in recordings with larger variability and more extreme measurement values.

In any case, applying imputation should be weighted against alternative methods to compensate for missing data, such as performing weighted or available-case analysis, or abstaining from analysis or decisions in case of incomplete data [35]. In this consideration, relevant factors include not only the possible error rates but also the understandability for clinical staff, the computational time [16], and whether complete data availability is needed for clinically used algorithms or for decision-making [36]. Last, the prevalence, duration, and nature of missing data should be taken into account. According to the classification of missing data as defined by Rubin [37], most of the tested techniques assumed data ‘missing completely at random’ (MCAR) and were also tested by randomly simulating missing data in the current study. However, MCAR assumptions may not always hold in clinical practice [38, 39]. Although technical disturbances such as connection issues are likely to occur completely at random, factors such as skin type or patient activities could systematically influence the likelihood of missing data related to sensor detachment or motion artefacts. In case the missingness is related to known factors and is not related to the signal characteristics of the vital parameter itself, data ‘missing at random’ (MAR) can be assumed. Furthermore, situations where the reasons for missing data are unknown or where missingness is associated with (pathological) vital sign abnormalities can occur, for example when measurements are disturbed by sweating in patients with fever or by motion artefacts related to delirium in deteriorating patients. In these cases, data is assumed to be ‘missing not at random’ (MNAR). As the performance of imputation techniques can be influenced in MAR and MNAR situations, as illustrated by the increased errors ranges found in data windows with larger variability or extreme vital sign levels, further investigation of the circumstances and possibilities to correct for these factors, for example by using accelerometry data, is of interest. Nevertheless, it should be realized that it will often be difficult to identify underlying reasons for missingness as context information is often lacking or cannot be objectified automatically. Therefore, it is recommended that the effects of imputation are validated in the intended care setting.

### Limitations and recommendations

To our knowledge, this is the first study that evaluated imputation techniques for wireless vital signs monitoring in a ward setting. The data used for simulation included many hours of recording but was obtained in a relatively small population including only two patient groups from one hospital. As vital signs characteristics vary between and within patient groups, this could specifically have influenced the results of the CBP method which relies on population data. To minimize the selection bias, we used random and repeated gap simulation and limited the number of simulation windows per patient. However, gap segments generated in the simulation iterations may have overlapped, in particular, for large gap lengths. Furthermore, gaps were only simulated in data segments with complete data to allow performance evaluation, and may therefore underrepresent situations where missing data is (most) likely to occur in real practice. Together, external validation of results in a larger dataset and for other patient groups is recommended, where MAR or MNAR scenarios are also explored in more detail. Besides, verification of the performance for other sensor systems is desired, taking into account the variable accuracy and different measurement techniques of wearable devices [40, 41].

By comparing estimations of the window slope and mean before and after imputation, we aimed to gain insights into the range of bias that can be expected when extracting signal features relevant for ward patient monitoring. Likewise, we explored possible consequences on clinical decision-making by evaluating changes in EWS points. However, as no standard guidelines for the analysis of continuous data in ward patients exist as of yet, these results are only illustrative. The effects were only investigated for single parameters, whereas a full EWS and other risk prediction models typically rely on multiple vital parameters and also include other clinical variables. Besides, the signal features and EWS points were only obtained in two-hour windows, while dynamic characteristics vary per vital parameter and per individual due to differences in underlying (patho)physiology. Last, the effect of imputation was only studied for a limited range of gap sizes and was not explored for windows with multiple gaps or other data sampling frequencies. Therefore, depending on the diagnostic aims and data characteristics, it might be relevant to verify the effects of imputation on other signal features or when using shorter or longer data windows. Likewise, it is recommended to evaluate the performance of imputation techniques for patterns of clinical interest, for example by exploring pathophysiological data or by comparing stable, linear, and non-linear trend patterns [19].

The current study only investigated a selection of imputation techniques for retrospective monitoring, while many other techniques for imputing missing data in physiological waveforms or data streams have been described [42]. Examples include Kalman-filters [19], Gaussian processes [15], probabilistic data recovery methods using data from related sensors [43], and neural networks [17]. Furthermore, we only investigated the performance of single imputation techniques, which by definition create bias and neglect variability of the missing values in risk models [35]. Methods that account for imputation uncertainty, such as multiple imputation or maximum likelihood methods, could be valuable to reduce bias in decision models [14, 38, 39]. Although the development and evaluation of these and other advanced imputation methods require in-depth analysis of missing data characteristics and relevant covariates—which was beyond the scope of this study—further investigation is highly recommended in future studies that aim to find the best imputation methods for a specific clinical decision model or for real-time monitoring. Likewise, it is of interest to investigate whether errors introduced by imputation methods can be predicted, for example, using historical signal characteristics, activity level, or prior signal quality. This knowledge may help to indicate the accuracy of imputed data and contribute to safe implementation. To encourage further investigation and development of imputation techniques, the dataset used in the current study is available to other researchers on request.

### Conclusion

Imputation of missing data periods in continuous vital signs recordings can be useful to facilitate data analysis for patient monitoring and risk modelling, but imputation errors vary strongly between cases and increase for larger gap sizes. Mean percentage errors differ between vital parameters and are highest for respiratory rate measurements. Although the studied imputation techniques showed overlapping error ranges, errors were structurally lowest for linear interpolation, followed by the cluster-based prognosis technique. Correspondingly, these techniques had the lowest impact on signal features and calculation of early warning scores, and are therefore recommended for retrospective imputation of vital signs measurements. In contrast, spline interpolation or a mean- or last-observation carried forward technique were associated with larger ranges of signal features bias compared to performing no imputation, and can therefore increase the uncertainty for risk modelling. Further investigation of factors influencing imputation errors and evaluation of (acceptable) risks for clinical decision-making is desired to promote safe implementation in clinical care.

### Supplementary Information

Below is the link to the electronic supplementary material.

Supplementary material 1 (PDF 426.5 kb)

Supplementary material 2 (PDF 135.9 kb)

Supplementary material 3 (PDF 410.0 kb)

Supplementary material 4 (PDF 459.3 kb)

## Abbreviations

°C: degree Celsius
(A)E: (Absolute) error
Bpm: beats per minute
Brpm: breaths per minute
CBP: cluster-based prognosis
EWS: early warning score
HR: heart rate
IQR: interquartile range
LI: linear interpolation
LOCF: last observation carried forward
MAE: mean absolute error
MCF: mean carried forward
MPE: mean percentage error
RR: respiratory rate
SD: standard deviation
SI: spline interpolation
SpO2: blood oxygen saturation
Temp: axillary temperature
