# Supplementary material for: Missing data imputation techniques for wireless continuous vital signs monitoring
Source: J Clin Monit Comput. 2023 Feb 2;37(5):1387–400. doi: 10.1007/s10877-023-00975-w (PMC9893204; doi:10.1007/s10877-023-00975-w)
Supplement: Supplementary file 1 — Supplementary material 1 (PDF 426.5 kb) [file 10877_2023_975_MOESM1_ESM.pdf]

Supplementary file 1. Cluster-based prognosis technique

Cluster-based prognosis technique

The cluster-based prognosis technique (CBP) aims to impute periods of missing vital signs data ('gaps') of a patient ('query patient') based on resembling data segments obtained in similar patients. Accordingly, this method is based on the assumption that vital signs patterns depend on patient profile and may therefore be predicted using patterns observed in patients with similar profiles. The CBP method was modified from a missing data imputation approach that was developed by Sun et. al. and used for mining of physiological data streams [1]. The CBP method involves three steps, including: (1) patient matching based on a population clustering model; (2) window matching; and (3) missing data imputation, as illustrated in Figure 11. The following sections specify the development of the clustering model and steps of the CBP method.

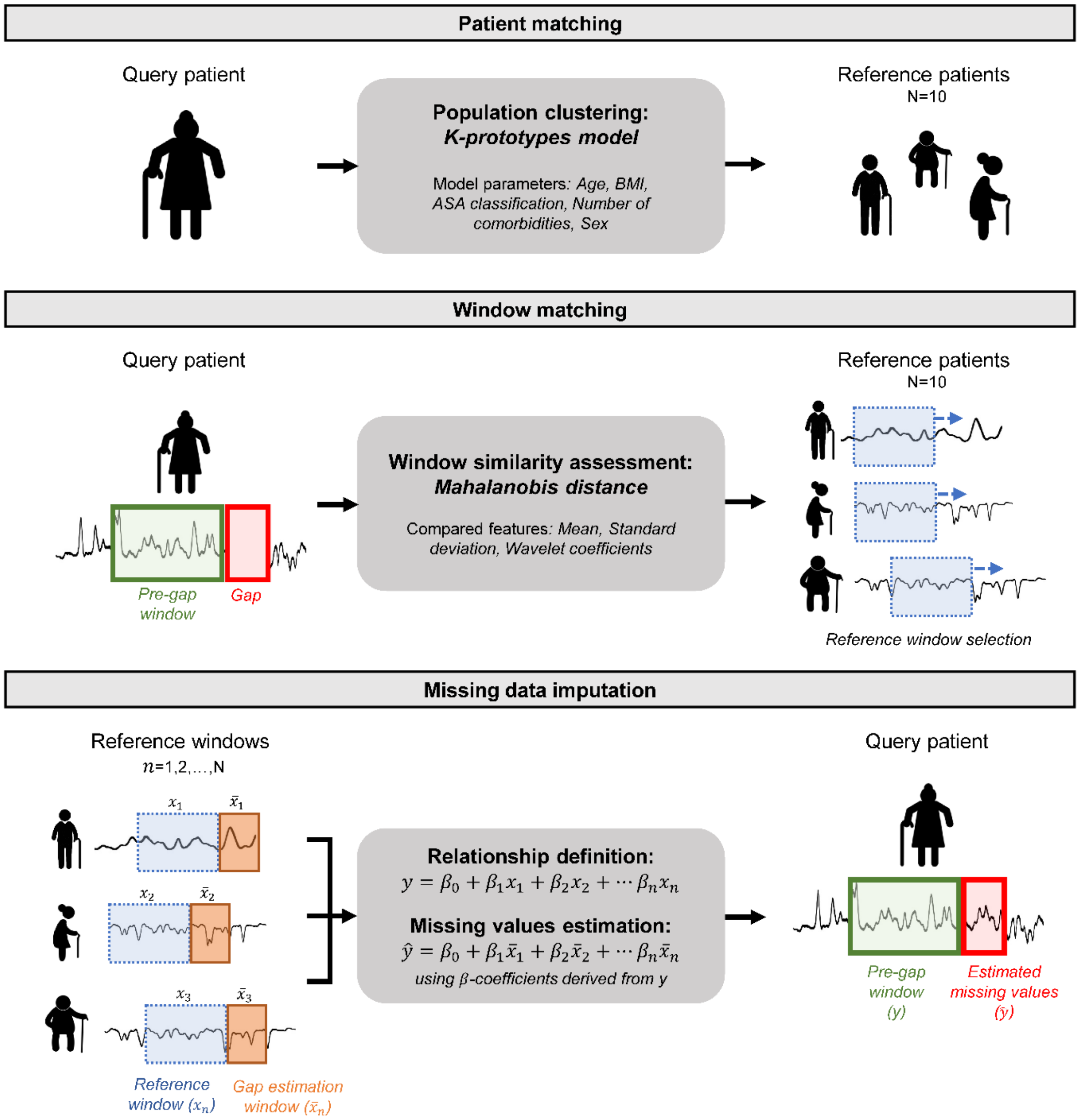

Fig. 11 Illustration of main steps of the cluster-based prognosis technique

Patient matching

The patient matching procedure is the first step of the CBP method, which aims to identify patients in a population that are most similar to the query patient. The similar patients are identified using a population clustering model, based on patient demographics. The population clustering model was developed using the previously described study database, including continuous vital signs recordings and patient demographics from 60 surgical ward patients. A total of 8 patients were excluded from the study database due to incomplete demographical data, resulting in 52 patients included for model development.

While Sun et. al. used a supervised learning-based approach involving labels provided by experts for population clustering, the CBP method employs an unsupervised learning-based clustering model which was developed using the K-prototypes algorithm. The K-prototypes algorithm was proposed by Huang et. al. and provides a combination of the well-known K-means and K-modes algorithms supporting clustering with numerical as well as categorical features. The K-prototypes algorithm was built following Huang's clusters centroid's initialization and gamma parameter selection method [2,3]. The optimal number of clusters was defined by assessing clustering quality through the average silhouette coefficient metric [4].

Model features that were explored included numerical parameters (*Age, Body mass index (BMI), American Society of Anesthesiologists (ASA) classification, Number of comorbidities*) and categorical parameters (*Sex, Type of surgery*). Numerical features were rescaled using z-score standardization. The K-prototypes algorithm was first built using all available model features and then optimized by removing features for which minimal contribution to the model was expected. For this aim, mutual information was calculated for each feature, describing the level of dependency between the feature and the cluster composition [5]. In addition, correlations between features were calculated using Pearson's and Spearman's correlation coefficient (numerical pair), Eta coefficient (numerical-categorical pair), or Cramer's V coefficient (categorical pair). Based on this correlation analysis, the parameters *Age* and *Type of surgery* were found to be correlated (p-value ≤ 0.05, Eta coefficient ≥ 0.7) which resulted in removal of *Type of surgery* from the model feature set as this feature had lowest mutual information. The mutual information of the remaining features that were used to build the final clustering model are shown in Table 2.

***Table 2** Mutual information of features in population clustering model*

Model feature	Mutual information
Sex	0.14
Age	0.39
Body mass index	0.14
ASA classification	0.04
Number of comorbidities	0.08

ASA: American Society of Anesthesiologists

After feature selection, global optimization was performed by repeatedly building a K-prototypes algorithm based model with different initial centroids in each of the 100 iterations. The model with highest average silhouette coefficient (0.5 (SD ± 0.2)) was selected as final population clustering model and used to identify clusters in the study population. The final clustering model resulted in two population clusters with significantly different patient characteristics, as described in Table 3.

***Table 3** Characteristics of population clusters*

Characteristics	Cluster 1 (N=18)	Cluster 2 (N=34)	P-value
**Sex**			<0.01
Male	16 (89%)	12 (35%)	
Female	2 (11%)	22 (65%)	
**Age (years)**	58 ± 9	77 ± 8	<0.01
**Body mass index (kg/m2)**	30 ± 6	24 ± 4	<0.01
**ASA classification**			<0.05
I	0 (0%)	0 (0%)	
II	13 (72%)	14 (41%)	
III	4 (22%)	17 (50%)	
IV	1 (6%)	3 (9%)	
**Number of comorbidities**	2 [1-3]	4 [3-5]	<0.01
**Type of surgery**			<0.01
Upper gastrointestinal cancer	17 (94%)	16 (47%)	
Hip fracture	1 (6%)	18 (53%)	

All values were reported as mean ± standard deviation, median [interquartile range], or number (percentage). ASA: American Society of Anesthesiologists

As a final step, the query patient's demographical features are provided as input for the developed clustering model to identify the 10 closest patients belonging to the query patient's identified cluster. These 10 patients are selected as 'reference patients' used in the window matching process.

Window matching

In the window matching process, the data segment ('reference window') that most resembled the data of the 60 min window prior to the gap ('pre-gap window') of the query patient was selected from the vital signs recordings for each reference patient. To select a reference window, the query patient's pre-gap window was compared with 60 min data windows from the concerning reference patient using a sliding window approach with a step length of 30 min. Comparison was performed after preprocessing of the vital signs data (see: study methods) and only within the same type of vital parameter. The windows were compared based on 12 features, including the signal mean, standard deviation, and top ten coefficients of the discrete wavelet transform using the Daubechies-4 wavelet. Window similarity was assessed using the distance between these features. While Sun et al. used a tailored distance metric based on supervised learning, the CBP method implemented the Mahalanobis distance to assess window similarity. To support missing data imputation in further steps, windows were only eligible to be selected as reference windows in case the period after the window with the same duration as the gap segment had data availability for at least 50% of the time. The window with highest similarity that met this data availability criterion was selected as reference window and used as anchor point to align the query and reference data. In case data availability was insufficient for the top five resembling windows, no window was selected for the corresponding reference patient.

Missing data imputation

To enable imputation of the missing data, the relationship between the samples in the query patient's pre-gap window and selected reference windows was described using the linear regression model: $y = \beta_0 + \beta_1 x_1 + \beta_2 x_2 + \cdots \beta_n x_n$, where y represents the 60 samples of the query patient's pre-gap window, x_i represent the 60 samples of the reference window of reference patient $i = 0,1, \dots n$. The regression parameters β_i, $i = 0,1, \dots n$ were solved using the least squares estimator. The resulting regression model was used to estimate the missing values

of the query patient, assuming that there is an equal relationship between the query patient data and the aligned reference patient data in the 60 min before the data gap and during the data gap. Accordingly, the data segment succeeding the reference window (‘Gap estimation window’) was selected in each reference patient data, adopting the same segment length as the query patient’s gap, and used as input in the model: $\hat{y} = \beta_0 + \beta_1\bar{x}_1 + \beta_2\bar{x}_2 + \cdots \beta_n\bar{x}_n$ where $\hat{y}$ represents the estimated missing values, $\bar{x}_i$ the gap estimation window of reference patient $i = 0,1, \dots n$, and β_i, $i = 0,1, \dots n$ the regression parameters derived from the previous regression model. In case x_i contained missing data values, these were replaced by the mean of the gap estimation window. In addition to Sun’s method, the estimated data values were filtered using a 4 min window-based median filter as this was also applied to vital signs data in the study database, and values were limited to the range between the last value prior and the first measurement after the data gap respectively to prevent the possibility of extreme values. Estimated data values that exceeded the upper or lower limit of this range were cut off and replaced by the corresponding limit value.

References

1. Sun J, Sow D, Hu J, Ebadollahi S (2010). A System for Mining Temporal Physiological Data Streams for Advanced Prognostic Decision Support. 2010 IEEE International Conference on Data Mining, pp. 1061–6. https://doi.org/10.1109/ICDM.2010.102

2. Huang Z (1997). Clustering large data sets with mixed numeric and categorical values. Proceedings of the 1st pacific-asia conference on knowledge discovery and data mining,(PAKDD), pp. 21–34.

3. Huang Z (1998) Extensions to the k-Means Algorithm for Clustering Large Data Sets with Categorical Values. Data Min Knowl Discov 2:283–304. https://doi.org/10.1023/A:1009769707641

4. Arbelaitz O, Gurrutxaga I, Muguerza J, Pérez JM, Perona I (2013) An extensive comparative study of cluster validity indices. Pattern Recognit 46:243–56. https://doi.org/10.1016/j.patcog.2012.07.021

5. Kraskov A, Stögbauer H, Grassberger P (2004) Estimating mutual information. Phys Rev E 69:066138. https://doi.org/10.1103/PhysRevE.69.066138
