# Supplementary material for: Missing data imputation techniques for wireless continuous vital signs monitoring
Source: J Clin Monit Comput. 2023 Feb 2;37(5):1387–400. doi: 10.1007/s10877-023-00975-w (PMC9893204; doi:10.1007/s10877-023-00975-w)
Supplement: Supplementary file 2 — Supplementary material 2 (PDF 135.9 kb) [file 10877_2023_975_MOESM2_ESM.pdf]

Supplementary file 2. Characteristics of included patients

Table 4 *Characteristics of included patients*

**Characteristics**	**Total**<br>(N=52)
**Sex**	
Male	28 (54%)
Female	24 (45%)
**Age (years)**	70 ± 13
**Body mass index (kg/m^2)**	26 ± 5
**ASA classification**	
I	0 (0%)
II	27 (52%)
III	21 (40%)
IV	4 (8%)
**Number of comorbidities**	3 [2-4]
**Type of surgery**	
Upper gastrointestinal cancer	33 (63%)
Hip fracture	19 (37%)

All values were reported as mean ± standard deviation, median [interquartile range], or number (percentage)
