# Supplementary material for: Missing data imputation techniques for wireless continuous vital signs monitoring
Source: J Clin Monit Comput. 2023 Feb 2;37(5):1387–400. doi: 10.1007/s10877-023-00975-w (PMC9893204; doi:10.1007/s10877-023-00975-w)
Supplement: Supplementary file 3 — Supplementary material 3 (PDF 410.0 kb) [file 10877_2023_975_MOESM3_ESM.pdf]

Supplementary file 3. *MAE_{gap}* for different levels of assessment window's mean and standard deviation

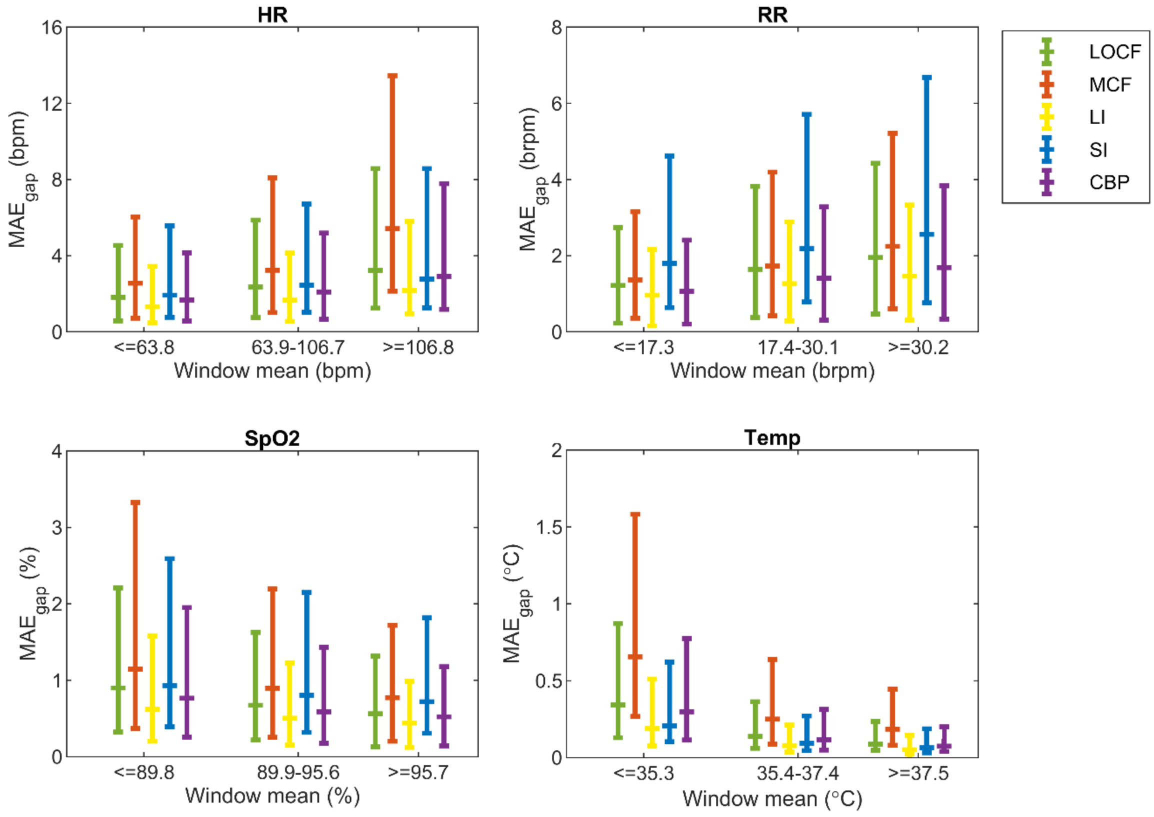

Fig. 12 *Mean Absolute Error (MAE_{gap}) observed for imputation of simulated missing data periods, clustered for assessment windows with 10% lowest mean value (left cluster), 10% highest mean value (right cluster) and remaining assessment windows (middle cluster). The MAE_{gap} is shown as median with interquartile range, and the horizontal axis reports the range of assessment window mean for each cluster. LOCF: last observation carried forward, MCF: mean carried forward, LI: linear interpolation, SI: spline interpolation, CBP: cluster-based prognosis, HR: Heart rate, RR: Respiratory rate, SpO2: blood oxygen saturation, Temp: Temperature*

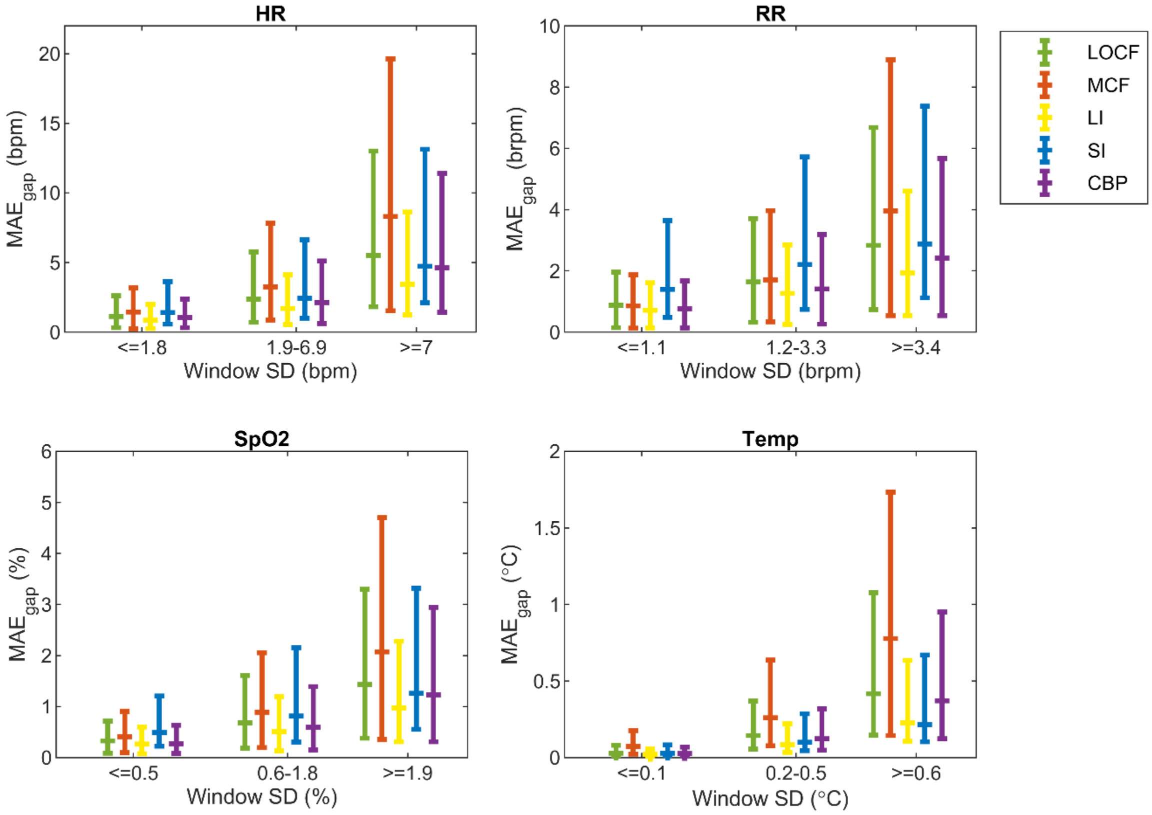

Fig. 13 *Mean Absolute Error (MAE_{gap}) observed for imputation of simulated missing data periods, clustered for assessment windows with 10% lowest standard deviation (left cluster), 10% highest standard deviation (right cluster) and remaining assessment windows (middle cluster). The MAE_{gap} is shown as median with interquartile range, and the horizontal axis reports the range of standard deviation for each cluster. SD: standard deviation, LOCF: last observation carried forward, MCF: mean carried forward, LI: linear interpolation, SI: spline interpolation, CBP: cluster-based prognosis, HR: Heart rate, RR: Respiratory rate, SpO2: blood oxygen saturation, Temp: Temperature*
