# Supplementary material for: Missing data imputation techniques for wireless continuous vital signs monitoring
Source: J Clin Monit Comput. 2023 Feb 2;37(5):1387–400. doi: 10.1007/s10877-023-00975-w (PMC9893204; doi:10.1007/s10877-023-00975-w)
Supplement: Supplementary file 4 — Supplementary material 4 (PDF 459.3 kb) [file 10877_2023_975_MOESM4_ESM.pdf]

Supplementary file 4. $AE_{2h\text{-}mean}$ and $AE_{2h\text{-}slope}$

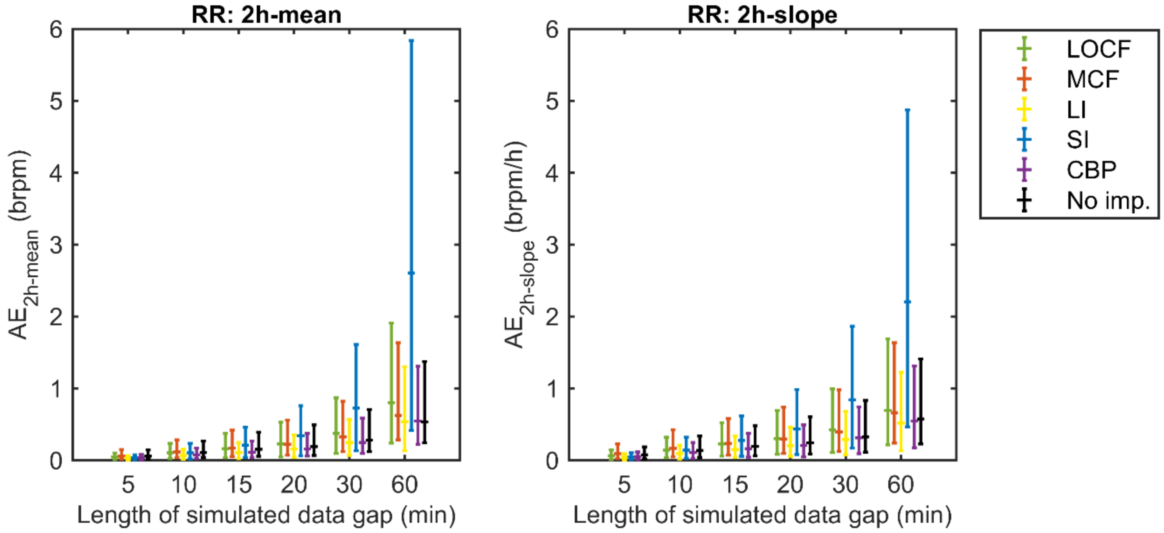

Fig. 14 *Absolute error of the mean value ($AE_{2h\text{-}mean}$) and the slope ($AE_{2h\text{-}slope}$) of the two-hour simulation window found for the respiratory rate (RR). The absolute error is shown as median with interquartile range for different imputation techniques and for the situation without imputation. LOCF: last observation carried forward, MCF: mean carried forward, LI: linear interpolation, SI: spline interpolation, CBP: cluster-based prognosis, No imp.: No imputation*

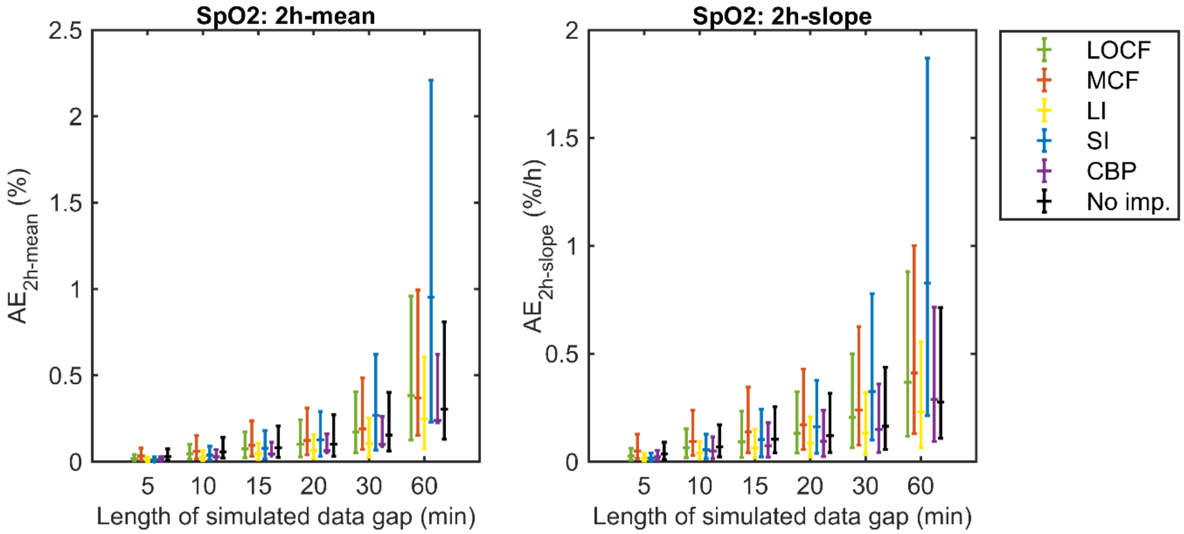

Fig. 15 *Absolute error of the mean value ($AE_{2h\text{-}mean}$) and the slope ($AE_{2h\text{-}slope}$) of the two-hour simulation window found for the blood oxygen saturation (SpO2). The absolute error is shown as median with interquartile range for different imputation techniques and for the situation without imputation. LOCF: last observation carried forward, MCF: mean carried forward, LI: linear interpolation, SI: spline interpolation, CBP: cluster-based prognosis, No imp.: No imputation*

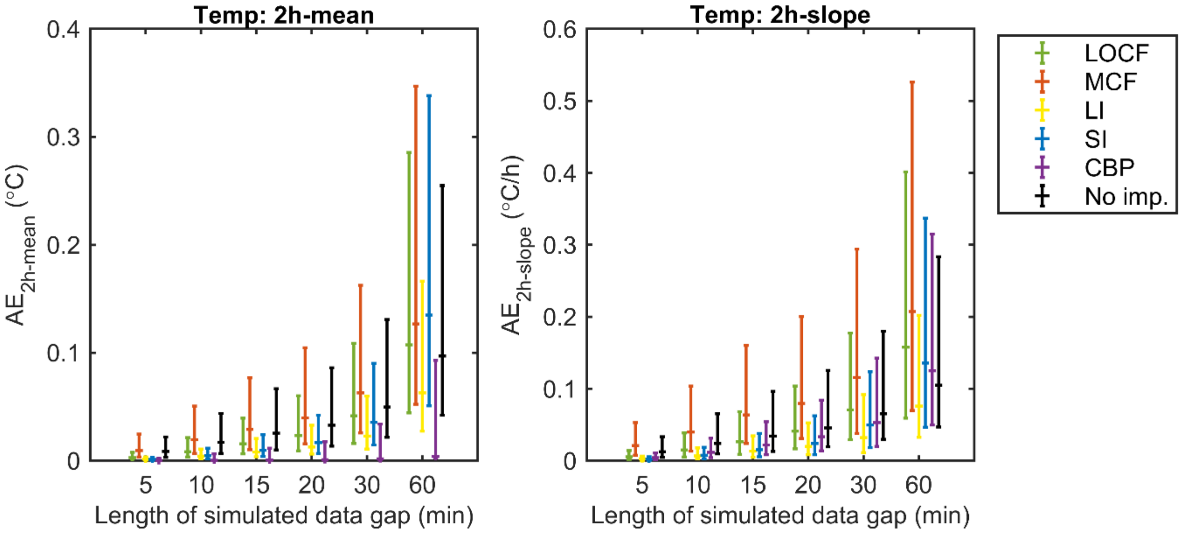

***Fig. 16** Absolute error of the mean value ($AE_{2h\text{-}mean}$) and the slope ($AE_{2h\text{-}slope}$) of the two-hour simulation window found for the temperature (Temp). The absolute error is shown as median with interquartile range for different imputation techniques and for the situation without imputation. LOCF: last observation carried forward, MCF: mean carried forward, LI: linear interpolation, SI: spline interpolation, CBP: cluster-based prognosis, No imp.: No imputation*
